# Investigation of Factors Influencing the Effectiveness of Deformable Nanovesicles for Insulin Nebulization Inhalation

**DOI:** 10.3390/pharmaceutics16070879

**Published:** 2024-06-29

**Authors:** Jinghan Yu, Yingying Meng, Zhiyang Wen, Yu Jiang, Yiyue Guo, Simeng Du, Yuling Liu, Xuejun Xia

**Affiliations:** 1 Beijing Key Laboratory of Drug Delivery Technology and Novel Formulations, State Key Laboratory of Bioactive Substance and Function of Natural Medicines, Department of Pharmaceutics, Institute of Materia Medica, Chinese Academy of Medical Sciences & Peking Union Medical College, Beijing 100050, China; yujinghan@imm.ac.cn (J.Y.); mengyingying@imm.ac.cn (Y.M.); wenzhiyang@imm.ac.cn (Z.W.); yu.jiang@wehandbio.com (Y.J.); dusimeng@imm.ac.cn (S.D.); 2 Beijing Wehand-Bio Pharmaceutical Co., Ltd., Beijing 102600, China; yiyue.guo@wehandbio.com

**Keywords:** nebulized inhalation, deformable nanovesicles, biomacromolecule, influencing factors

## Abstract

Nebulized inhalation offers a noninvasive method for delivering drugs to treat both local respiratory and systemic diseases. In this study, insulin was used as a model drug to design a series of deformable nanovesicles (DNVs) with key quality attributes, including particle size, deformability, and drug load capacity. We investigated the effects of these properties on aerosol generation, macrophage phagocytosis, and bloodstream penetration. The results showed that deformability improved nebulization performance and reduced macrophage phagocytosis, benefiting local and systemic delivery. However, the advantage of DNVs for transmembrane penetration was not evident in the alveolar epithelium. Within the size range of 80–490 nm, the smaller the particle size of IPC-DNVs, the easier it is to evade clearance by macrophages and the more effective the in vivo hypoglycemic efficacy will be. In the drug load range of 3–5 mg/mL, a lower drug load resulted in better hypoglycemic efficacy. The area above the blood glucose decline curve with time (AAC) of nebulized DNVs was 2.32 times higher than that of the insulin solution, demonstrating the feasibility and advantages of DNVs in the pulmonary delivery of biomacromolecule drugs. This study provides insights into the construction and formulation optimization of pulmonary delivery carriers.

## 1. Introduction

The application of nanotechnology to the design of drug delivery systems has opened a unique avenue for disease treatment. These systems improve the solubility of poorly soluble drugs, protect drugs from physiological environmental interference and degradation, and transport drugs precisely and effectively to disease sites and therapeutic targets. Consequently, they significantly improve drug efficacy and reduce adverse reactions [1,2,3]. Compared with other nanocarriers, lipid nanovesicles are widely used in drug delivery systems because of their good safety profile and ability to enhance drug bioavailability [4]. They are also the only nanostructures approved and marketed for inhalation drug delivery [5]. As a special type of lipid nanovesicles, deformable nanovesicles (DNVs) introduce edge-activating surfactants into their bilayers, thereby disrupting the balance of the phospholipid bilayer. This imparts deformability on the nanovesicles, allowing them to squeeze through biological mucosal barriers narrower than their size. Consequently, they facilitate the transmembrane delivery of various small molecules and poorly permeable macromolecular drugs [6,7,8]. DNVs have been widely applied in drug delivery via transdermal, oral, parenteral, and pulmonary routes [9].

Nebulized inhalation is a highly attractive alternative noninvasive drug delivery method. Given the unique physiological attributes of the lungs, such as large absorption area, high vascularization, low metabolic enzyme activity, and absence of the liver’s first-pass effect [10,11], nebulized inhalation is effective for treating local respiratory diseases [12,13,14] by increasing drug concentration at the disease site and minimizing systemic side effects. It also opens new avenues for delivering drugs with low oral bioavailability, such as protein and peptide drugs like insulin [15,16], organophosphorus hydrolase [17], and poorly permeable BCS class III drugs like atenolol [18].

Despite the advantages of nebulized inhalation, clinical translation remains limited [19]. In addition to being constrained by the physicochemical properties of delivered drugs, this delivery method faces several challenges. Firstly, the drug must be converted into an aerosol with an appropriate aerodynamic particle size for deposition at the target site. Particles with a mass median aerodynamic diameter (MMAD) of 1–5 μm are optimal for deep lung deposition [2], dependent on the nebulizer device and drug formulation [20]. Ongoing advancements in nebulizer devices provide patients with more options, allowing them to choose a suitable nebulizer based on their condition [21]. Notably, formulation factors such as the viscosity and surface tension of the nebulized solution affect nebulization performance and behavior [22]. Secondly, after deposition at the target site, the drug must penetrate various barriers to exert its therapeutic effects on systemic diseases. These barriers include the surfactant layer (mainly composed of phospholipids) on the alveolar surface [23] and the monolayer of alveolar epithelial cells. Conversely, for local respiratory treatments, entering the circulation can reduce the drug concentration at the target site and cause systemic adverse reactions [24]. In addition, phagocytic clearance by alveolar macrophages must also be considered, as many macrophages patrolling the alveoli will constantly attempt to engulf and digest deposited insoluble particles [25].

Compared with other administration routes, nebulized inhalation faces the dual challenges of aerosol generation and navigating the complex physiological environment of the lungs. This necessitates advanced carrier construction for pulmonary delivery of biomacromolecule drugs. We already know the advantages of DNVs for drug transmembrane delivery, and researchers have attempted to use DNVs for the pulmonary delivery of small-molecule drugs such as beclomethasone dipropionate [9], salbutamol sulfate [26], and paclitaxel [27] via nebulized inhalation. However, current studies focus only on small-molecule drugs and their compatibility with various nebulizers, without addressing the importance of deformability in pulmonary delivery or systematically investigating the nebulization process and in vivo fate. Furthermore, while existing studies have demonstrated the feasibility of using DNVs for the pulmonary delivery of small-molecule drugs, there have been no reports of their use for the pulmonary delivery of biomacromolecule drugs. Additionally, certain physicochemical properties of nanoparticles, like particle size [28], surface charge [29], membrane fluidity [30], and surface modification [31], affect their interaction with pulmonary physiological barriers following inhalation. This suggests that studying other key quality attributes of DNVs besides deformability and their impact on the nebulization process and in vivo fate may also be crucial for optimizing their design as carriers for the pulmonary delivery of biomacromolecule drugs.

In our previous study, we attempted to deliver insulin across the oral mucosa using DNVs, confirming the superiority of deformable liposomes for transmucosal delivery of biomacromolecule drugs [32]. In this study, we focus on insulin-loaded deformable nanovesicles to investigate the factors and feasibility of using these nanovesicles for insulin aerosolized inhalation delivery, aiming to provide insights into constructing and optimizing delivery vehicles for biopharmaceuticals targeting the lungs. Firstly, we formulated a series of deformable nanovesicles with insulin, varying in critical quality attributes such as particle size, deformability, and drug loading, and characterized their quality attributes. Subsequently, we assessed these nanovesicles based on three aspects crucial for aerosolized inhalation delivery of biopharmaceuticals: aerosol generation, macrophage phagocytosis, and penetration into the bloodstream to exert pharmacological effects. The evaluation methods included: (1) assessing the aerosol dynamic characteristics of insulin-loaded deformable nanovesicles using a vibrating mesh nebulizer; (2) investigating the uptake characteristics of these nanovesicles by RAW264.7 macrophages at the cellular level; and (3) evaluating the in vivo pharmacological efficacy of different formulations of insulin-loaded deformable nanovesicles in an SD rat tracheal instillation model, with blood glucose reduction as a measure of systemic penetration. Finally, we established a nose-only exposure nebulized inhalation model in SD rats to compare the efficacy of nebulized deformable vesicles and insulin solution, verifying the feasibility of using DNVs for insulin nebulized inhalation delivery in vivo. 

## 2. Materials and Methods

### 2.1. Materials

Recombinant human insulin (28.8 IU/mg) was purchased from Tonghua Dongbao Pharmaceutical Co., Ltd. (Tonghua, China). Soybean phospholipids (S75) were obtained from Shanghai Taiwei Pharmaceutical Co., Ltd. (Shanghai, China). Tween 20 was purchased from Nanjing Well Pharmaceutical Group Co., Ltd. (Nanjing, China). Sodium cholate was obtained from Anhui Chem-Bright Bioengineering Co., Ltd. (Huaibei, China). Trehalose was a gift from Pfanstiehl (Waukegan, IL, USA) and lactose was procured from Hunan Erkang Pharmaceutical Co., Ltd. (Changsha, China). d-Mannitol was purchased from Sigma-Aldrich (St. Louis, MO, USA), and FITC-labeled insulin was obtained from Dalian Meilun Biotechnology Co., Ltd. (Dalian, China). Mounting medium with DAPI for fluorescence quenching was sourced from Shanghai Beyotime Biotechnology Co., Ltd. (Shanghai, China). All reagents were of analytical or chromatographic grade. A handheld vibrating mesh nebulizer (M105) was purchased from Jiangsu Yuyue Medical Equipment and Supply Co., Ltd. (Yancheng, China).

### 2.2. Animals

SPF-grade male SD rats were purchased from Beijing HFK Bioscience Co., Ltd. (Beijing, China). All rats were raised at the Institute of Materia Medica, Chinese Academy of Medical Sciences and Peking Union Medical College (Beijing, China). The experiments were performed with the approval of the Animal Care and Welfare Committee of Institute of Materia Medica, Chinese Academy of Medical Sciences and Peking Union Medical College.

### 2.3. Preparation of Deformable Nanovesicle Based on Insulin–Phospholipid Complexes (IPC-DNVs) 

IPC-DNVs were prepared using a thin-film hydration method described in our previous study [32]. Briefly, a certain amount of Lipoid S75 (600 mg) and insulin (60 mg) were separately dissolved in dichloromethane (54 mL) and 0.1% trifluoroacetic acid–methanol (6 mL) in a round-bottom flask. After mixing, the solution was rotated at 37 °C to evaporate and form IPCs, followed by vacuum drying for 2 h to remove residual organic solvents. Appropriate amounts of IPCs, Lipoid S75 (600 mg), and Tween 20 (400 mg) were dissolved in dichloromethane to form a clear solution, which was evaporated at 37 °C using a rotary evaporator to obtain a lipid film. The resulting film was suspended in phosphate-buffered saline (PBS, pH 7.4) containing sodium cholate (100 mg) and rotated at 80 rpm for 30 min at 37 °C. After cooling, IPC-DNVs underwent four cycles of sonication (1 min each time), followed by uniformization through a 0.22 μm polycarbonate membrane. The final concentrations were as follows: Lipoid S75 was 60 mg/mL, Tween 20 was 20 mg/mL, sodium cholate was 5 mg/mL, and insulin was 3 mg/mL.

#### 2.3.1. Preparation of IPC-DNVs with Different Particle Sizes

We employed the freeze-drying method previously used to obtain IPC-DNVs with different particle sizes by adjusting the amount and type of cryoprotectant [33]. Briefly, 8% cryoprotectant was dissolved in IPC-DNV solution (0.5 mL), and PBS was added to dilute the solution to 1 mL. The freeze-drying procedure was as follows: first, the sample plate was cooled to −45 °C at a rate of 20 °C/h and maintained for 3 h. Then, the plate temperature was increased to −25 °C at a rate of 5 °C/h and held for 10 h. Subsequently, it was slowly heated to −10 °C for 3 h, then to 0 °C for 3 h, and finally heated to 10 °C at a rate of 5 °C/h for 3 h. Finally, the mixture was returned to room temperature and allowed to stand for 3 h. The freeze-dried IPC-DNVs were dispersed in distilled water to their original volume to form liquid IPC-DNVs at an insulin concentration of 3 mg/mL. By varying the amount and type of cryoprotectant (no cryoprotectant, 8% trehalose:lactose = 4:1, 5% trehalose:lactose = 4:1, 8% mannitol), IPC-DNVs with target sizes of 80 nm, 150 nm, 230 nm, and 490 nm were prepared.

#### 2.3.2. Preparation of IPC-DNVs with Different Deformability

With a fixed particle size of approximately 80 nm, we obtained IPC-DNVs with different deformability by adjusting the amount of edge activators (0%, 1.8%, and 2.5%).

#### 2.3.3. Preparation of IPC-DNVs with Different Drug Load

Using a fixed particle size of approximately 80 nm and 2.5% edge activator, we obtained IPC-DNVs with drug loads of 3, 4, and 5 mg/mL by varying the amount of insulin (60, 80, and 100 mg).

### 2.4. Characterization

#### 2.4.1. Determination of Size and Zeta Potential

The average particle size and zeta potential of the different IPC-DNVs were determined via dynamic light scattering using a laser particle size analyzer (PSS, NICOMP™ 380ZLS, Santa Clara, CA, USA). Prior to the assay, samples were diluted 10-fold with distilled water.

#### 2.4.2. Entrapment Efficiency (EE)

Ultrafiltration centrifugation was used to determine the drug entrapment efficiency of IPC-DNVs. First, 0.5 mL of IPC-DNVs were diluted to 25 mL with 0.01% TFA-methanol solution to measure the insulin content, representing the total insulin content. IPC-DNVs (1 mL) were placed in ultrafiltration tubes (Amicon Ultra-4 centrifuge set, 100 K NMWL; Millipore, Burlington, MA, USA) and centrifuged at 4000 rpm for 20 min to separate the free and entrapped insulin in the IPC-DNVs, after which the ultrafiltrate was collected. Subsequently, the ultrafiltration tubes were washed three times with PBS at 2500 rpm for 5 min to collect the ultrafiltrates. The insulin content in the ultrafiltrate was determined, and the sum of the insulin contents in the two-step ultrafiltrate was taken as the free insulin content. Insulin content was determined through high performance liquid chromatography (HPLC), using an Agilent Technologies 1200 series HPLC system (Agilent, Santa Clara, CA, USA) equipped with a 300SB-C18 column (4.6 × 250 mm, 5 μm, Agilent). The assay conditions were as follows: 0.2 M phosphate buffer/acetonitrile (74:26, *v*/*v*), flow rate: 1 mL/min, UV detection: 214 nm, injection volume: 20 μL, column temperature: 40 °C, solvent: 0.01% TFA-methanol solution. EE was calculated using the following formula:

(1) $$EE=\left(\frac{w_{total-insulin}-w_{free-insulin}}{w_{total-insulin}}\right)\times 100\%$$

where *W_total-insulin_* is the total insulin content in the IPC-DNVs and *W_free-insulin_* is the free insulin content.

#### 2.4.3. Deformability

The deformability index of the IPC-DNVs were determined using a stainless-steel pressure filtration device. The IPC-DNVs were extruded through a 50 nm polycarbonate membrane using a syringe at a constant pressure of 0.45 MPa applied by a syringe. The weight loss method was employed to calculate the weight of the liquid that flowed out within 5 min. As mentioned earlier, the particle size of the extruded IPC-DNVs was measured using a laser particle size analyzer. The deformability index of the IPC-DNVs was calculated using the following formula:

(2) $$DI=\;J\times {\left(\frac{r_{v}}{r_{p}}\right)}^{2}$$

where *J* is the weight of the outflow liquid, *r_v_* is the particle size of the extruded IPC-DNVs, and *r_p_* is the aperture of the filter membrane (50 nm).

### 2.5. Determination of Aerodynamic Characteristics

MMAD, fine particle dose (FPF), and geometric standard deviation (GSD) of IPC-DNVs were measured using a next-generation impactor (NGI) (Beijing Huironghe Technology, HRH-ZJQ-160, Beijing, China). Briefly, the NGI host was placed in a 4 °C freezer and pre-cooled for 90 min before use. Then, 3 mL of IPC-DNVs with different properties were added to the nebulizer’s medication cup. The nebulizer was connected to the NGI host and an air pump, and the nebulization process was initiated. Post-nebulization, IPC-DNVs were eluted with PBS at each stage, and the eluent from each stage was diluted five times with 0.01% TFA-methanol solution. The insulin content was determined using HPLC as described in Section 2.4.2, and the data were analyzed using the Inhalant evaluation data analysis software (version: 2.3.2.5).

### 2.6. Uptake by RAW 264.7 Cells

#### 2.6.1. Preparation of FITC-IPC-DNVs

The preparation method was the same as that described in Section 2.3, with part of the insulin replaced by FITC-labeled insulin, and the entire operation was conducted in the dark.

#### 2.6.2. Uptake Study

RAW264.7 cells are mouse mononuclear macrophages derived from tumors induced by the Abelson murine leukemia virus in male mice. They are commonly used as models to study cellular uptake. For this study, RAW264.7 cells in the log growth phase were prepared in a cell suspension, which was seeded in 12-well plates at a density of 2 × 10^5^ cells per well and cultured in a cell incubator for 24 h. IPC-DNVs with different properties were diluted to 100 μg/mL with complete medium (10% FBS + DMEM), and added to the wells with adhered cells. Subsequently, 12-well plates were incubated in a cell culture incubator at 37 °C under 5% CO_2_ for 4 h. The medium containing the fluorescent drug was discarded, and the wells were washed three times with pre-cooled PBS. Next, 1 mL of 4% paraformaldehyde was added to each well, and the cells were fixed at room temperature for approximately 15 min. The cells were washed three times with PBS, and 400 μL of antifade mounting medium containing DAPI was added to each well. Cellular uptake was qualitatively observed using an inverted fluorescence microscope (BiOTek, CYTATION/5, Santa Clara, CA, USA). Another set of cells was subjected to discard the medium and wash three times with pre-cooled PBS after incubation with the fluorescent drug, and cells from each well were collected by scraping. The cell suspension was centrifuged at 350× *g* at 4 °C for 5 min, the supernatant was discarded, and 500 μL PBS was added to re-suspend the cells. The mean fluorescence intensity (MFI) of FITC was measured in 10,000 cells in each group using flow cytometry (BD Biosciences, BD FACSCelesta, San Jose, CA, USA). 

### 2.7. Hypoglycemic Effect In Vivo

#### 2.7.1. Intratracheal Instillation Administration

SD rats (*n* = 3) were fasted overnight but allowed free access to water. Tail blood samples were collected to measure the fasting blood glucose levels. Rats were then anesthetized with 50 mg/kg Zoletil 50 (Virbac, Carros, France). After securing the rats on a surgical board, a horizontal incision was made in the neck, and the tissue was dissected to expose the trachea. The surgical board was positioned at 80° from the tabletop. Using a 1 mL syringe, 50 μL of IPC-DNVs with different properties (diluted with PBS before use) were rapidly injected between the fifth and sixth tracheal rings below the thyroid cartilage. After injection, the rats were placed vertically and rotated vertically for 1 min to ensure uniform distribution of the drug solution. Subsequently, the rats were positioned at a 30° angle, and tail blood glucose was measured every 30 min for 5 h following tracheal administration to assess changes in blood glucose levels. Simultaneously, the blank deformable vesicle group (*n* = 3) and the subcutaneous insulin solution (3 mg/mL) group (*n* = 3, 0.5 IU/Kg) were established. The relative pharmacological bioavailability (F) of insulin after intratracheal instillation was calculated as follows:

(3) $$F=\frac{{AAC}_{tracheal}\times {Dose}_{s.c.}}{{AAC}_{s.c.}\times {Dose}_{tracheal}}\times 100\%$$

where *AAC_tracheal_* and *AAC_s.c._* refer to the area above the blood glucose decline curve over time in the intratracheal instillation and subcutaneous injection groups, respectively, and *Dose_s.c._* and *Dose_tracheal_* refer to the dose of insulin injected subcutaneously and tracheally, respectively.

#### 2.7.2. Nebulized Inhalation Administration

**Figure 1:**
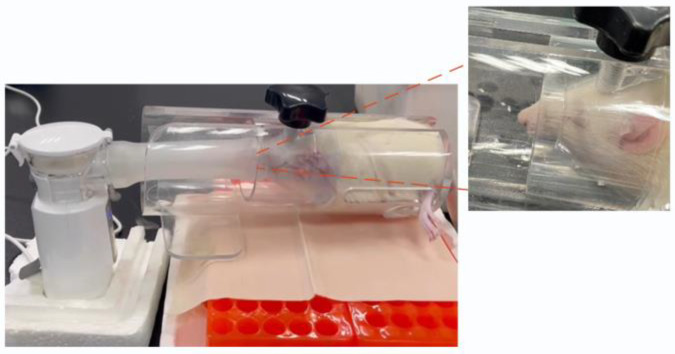
Nose-only exposure inhalation devise.

SD rats (*n* = 3) were fasted overnight but allowed free access to water. Tail blood samples were collected to measure the fasting blood glucose levels. Referring to the literature on the design of nose-only exposure devices [34], a simple rat nasal exposure fixation device was constructed by connecting plastic tubing to a rat holder (Figure 1). The nose-only exposure device features a funnel-shaped design at the front end, tailored to accommodate the pointed and protruding nose of rats. This design allows for rat immobilization while exposing the nose to aerosols through the funnel hole. The rats were placed in the fixation device and the tubing was connected to a nebulizer. Insulin saline solution (3 mg/mL) and IPC-DNVs (3 mg/mL) were added separately to a nebulizer medication cup. Nebulization was then initiated. After nebulization, the rats were released from the fixation device, and tail blood glucose was measured every 30 min for 5 h following aerosol inhalation to assess changes in blood glucose levels. Simultaneously, the blank deformable vesicle group (*n* = 3) was nebulized.

### 2.8. Statistical Analysis

Data are expressed as the mean ± standard deviation of three independent experiments using one-way analysis of variance. GraphPad Prism 8 software was used for plotting, and *t*-tests were used to determine statistically significant differences between groups. A *p*-value less than 0.05 was considered statistically significant (* *p* < 0.05, ** *p* < 0.01, *** *p* < 0.001).

## 3. Results

### 3.1. Preparation and Characterization of IPC-DNVs with Different Properties

In this study, IPC-DNVs with different deformability, particle sizes, and drug load were prepared by adjusting the amount of the edge activators, cryoprotectant, and insulin. The particle size, zeta potential, and entrapment efficiency of the IPC-DNVs were measured to evaluate their quality. Results are shown in Table 1, Table 2 and Table 3.

The IPC-DNVs obtained from the original prescription had edge activators amount of 2.5%, a particle size of 83.87 ± 0.71 nm, and a drugload of 3 mg/mL. By adjusting the dosage of the edge activator (0%, 1.8%, and 2.5%), we obtained IPC-DNVs with deformability index of 6.56 ± 0.66, 29.75 ± 0.28, and 39.30 ± 0.99 μg/cm^2^/s, respectively. The deformability index increased with the number of edge activators, indicating an increase in deformability. There were no apparent differences observed in particle size, zeta potential, or entrapment efficiency among the deformable groups, except that the entrapment efficiency of IPC-DNVs in the group with 0% edge activator dosage was slightly lower than that in the original prescription group. By adding different amounts and types of lyophilized protective agents, we obtained IPC-DNVs with different particle sizes (target particle size of 80 nm, 150 nm, 230 nm, and 500 nm, and actual particle size of 84 nm, 151 nm, 239 nm, and 478 nm). There were no apparent differences observed across the various particle size groups. By changing the amount of insulin (60, 80, and 100 mg), we obtained IPC-DNVs with different amount (3, 4, and 5 mg/mL). There were no apparent differences observed in the particle size, zeta potential, or entrapment efficiency of the IPC-DNVs among the drug-loading groups. We used the different properties of the IPC-DNVs prepared above to conduct subsequent in vitro and in vivo evaluations.

### 3.2. Impact of Different Properties on the Aerodynamic Characteristics of Aerosols Generated by Nebulization of IPC-DNVs

**Figure 2:**
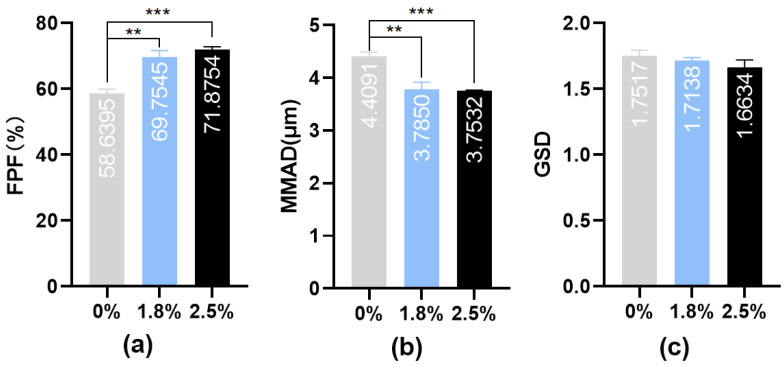
(**a**) FPF, (**b**) MMAD, and (**c**) GSD resulting from nebulization of IPC-DNVs with different edge activators amount (0%, 1.8%, 2.5%) nebulized by Yuwell mesh nebulizer. (*n* = 3). ** *p* < 0.01, *** *p* < 0.001.

**Figure 3:**
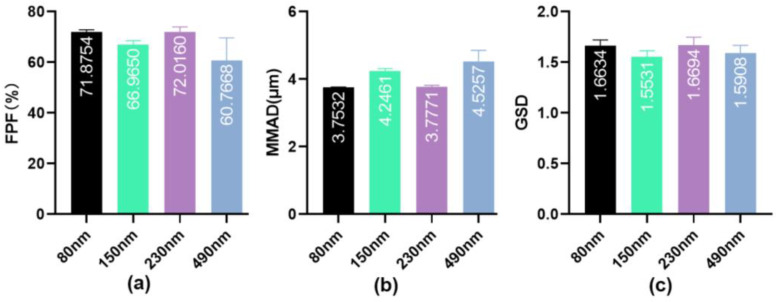
(**a**) FPF, (**b**) MMAD, and (**c**) GSD resulting from nebulization of IPC-DNVs with different particle sizes (80, 150, 230, 490 nm) nebulized by Yuwell mesh nebulizer. (*n* = 3).

**Figure 4:**
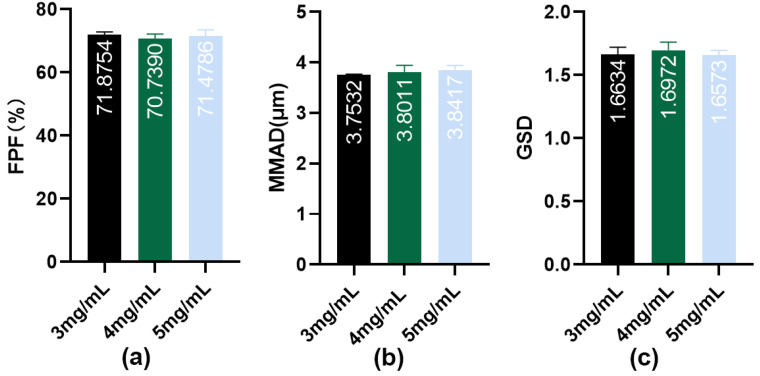
(**a**) FPF, (**b**) MMAD, and (**c**) GSD resulting from nebulization of IPC-DNVs entrapping increasing concentration of insulin (3, 4, 5 mg/mL) nebulized by Yuwell mesh nebulizer. (*n* = 3).

Aerosol generation is a key feature that distinguishes inhalation formulations from other formulations. Drug solutions are processed by a nebulizer into inhalable aerosols that require appropriate aerodynamic characteristics for deposition at the target site. The cascade impactor method is recommended by the European Pharmacopoeia as the “gold standard” for evaluating inhalation formulations. Aerosol particles are drawn into the impactor at an airflow rate of 15 L/min and pass through multiple stages, simulating realistic lung deposition [35]. To evaluate the in vitro nebulization performance of IPC-DNVs with different properties, their aerodynamic characteristics were determined through NGI using a handheld vibrating mesh nebulizer. The results are shown in Figure 2, Figure 3 and Figure 4 (The actual deposition percentages of insulin at each NGI stage can be found in Figures S1–S3.).

The NGI data of different deformable IPC-DNVs showed that the introduction of deformability optimized the atomization performance of the IPC-DNVs (FPF increased, while MMAD decreased), and the group with 0% edge activator amount (the lowest deformability) showed significant differences in FPF and MMAD compared with those of the 1.8% and 2.5% groups. However, there was no significant difference in FPF and MMAD when the amount of edge activators was in the range of 1.8–2.5%. In contrast, the NGI data of the IPC-DNVs for different particle size groups indicated that the particle size may be independent of the aerodynamic particle size of the aerosol generated by the nebulizer. The aerodynamic characteristics of the IPC-DNVs in the different insulin load groups were not significantly different, suggesting that variations in insulin load do not significantly affect atomization performance within a certain range.

### 3.3. Impact of Different Properties on the Uptake of IPC-DNVs by RAW264.7 Cells

#### 3.3.1. Qualitative Cellular Uptake Studies

**Figure 5:**
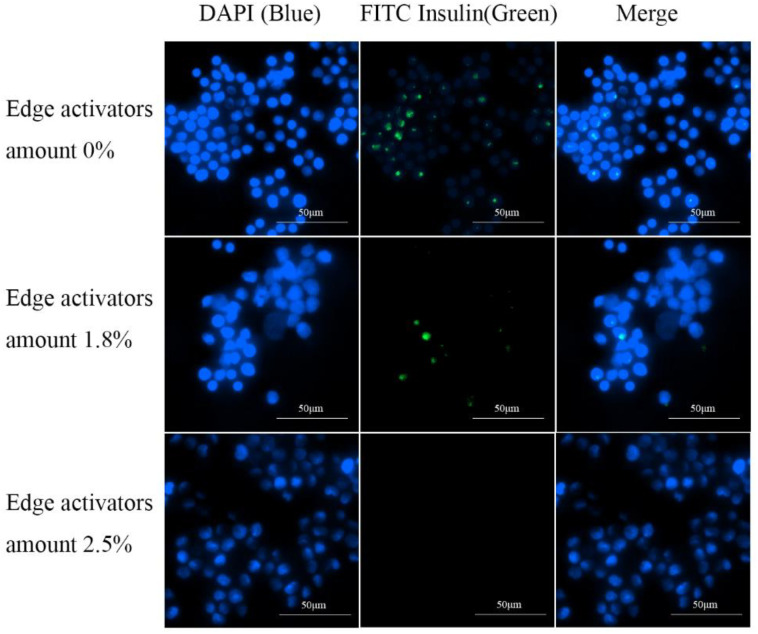
Fluorescence microscopy imaging of RAW264.7 cells uptaking IPC-DNVs with different edge activators amount (0%, 1.8%, 2.5%).

**Figure 6:**
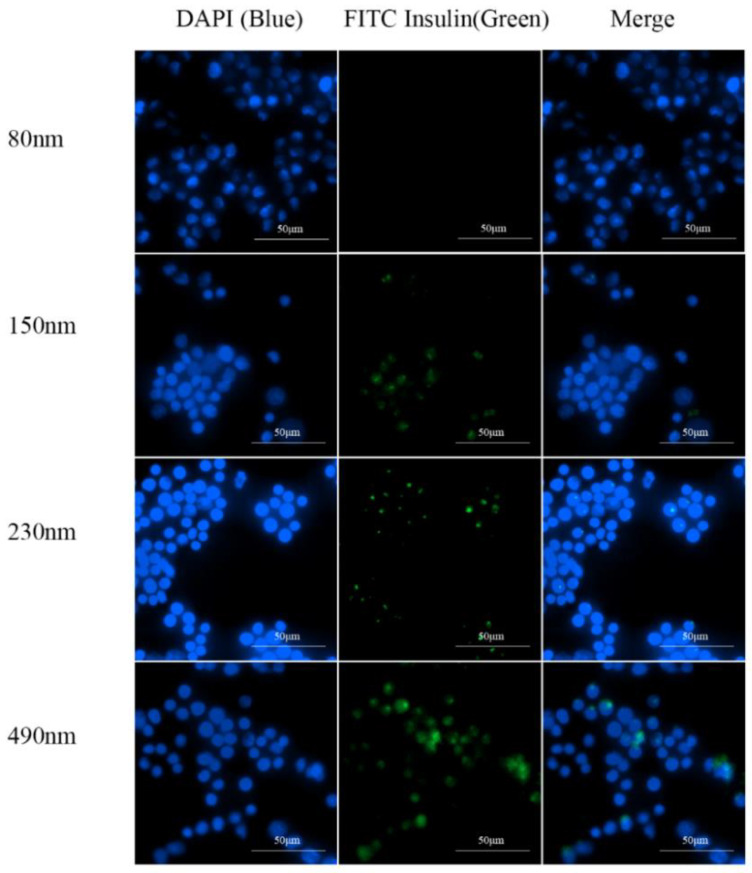
Fluorescence microscopy imaging of RAW264.7 cells uptaking IPC-DNVs with different particle sizes (80, 150, 230, 490 nm).

**Figure 7:**
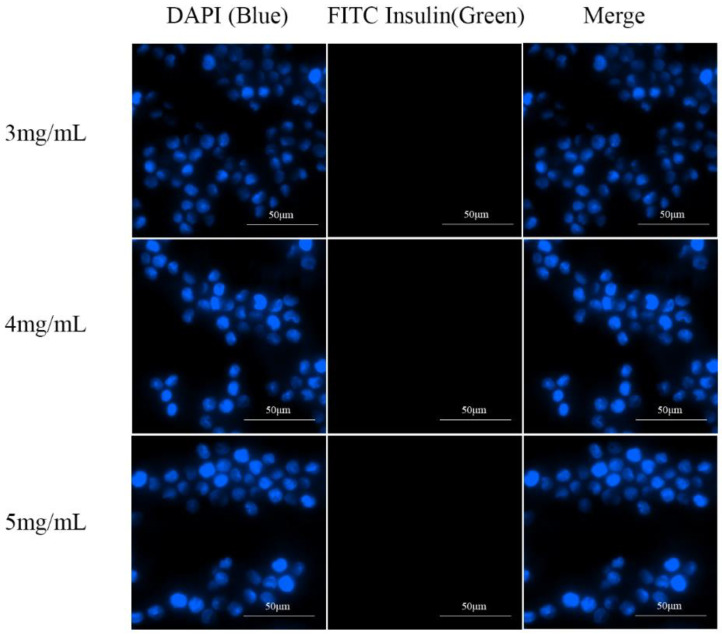
Fluorescence microscopy imaging of RAW264.7 cells uptaking IPC-DNVs entrapping increasing concentration of insulin (3, 4, 5 mg/mL).

Macrophages in the alveoli are an important barrier to the clearance of drugs entering deep into the lungs [36]. Except for drugs targeted inside macrophages [5], most pulmonary drugs must avoid alveolar macrophage uptake. To investigate key factors influencing IPC-DNV uptake by macrophages, we used RAW264.7 mouse mononuclear macrophages for in vitro uptake experiments with IPC-DNVs of different properties. The uptake results were captured using fluorescence microscopy and are shown in Figure 5, Figure 6 and Figure 7.

We observed an interesting phenomenon in the uptake experiments for the different deformability groups. Surprisingly, when the amount of the edge activator was 0% (the lowest deformability), the intracellular green fluorescence was the strongest, and the IPC-DNVs were the most ingested. As the IPC-DNVs became more deformable, the intracellular green fluorescence gradually decreased, indicating that fewer IPC-DNVs were taken up by the macrophages. These results suggest that the introduction of deformability facilitated the avoidance of macrophage clearance by IPC-DNVs. Moreover, in the uptake experiments with different particle size groups, no green fluorescence was observed in RAW264.7 cells co-incubated with IPC-DNVs in the 80 nm group, indicating no obvious uptake phenomenon. However, with the increase in vesicle size, green fluorescence in cells increased and was enhanced, and the uptake of IPC-DNVs into RAW264.7 cells was the highest in the 490 nm group. This suggests that particle size had a apparent effect on the uptake of IPC-DNVs into RAW264.7 cells. No obvious cellular uptake of IPC-DNVs was observed in the different drug load groups.

#### 3.3.2. Quantitative Cellular Uptake Studies

**Figure 8:**
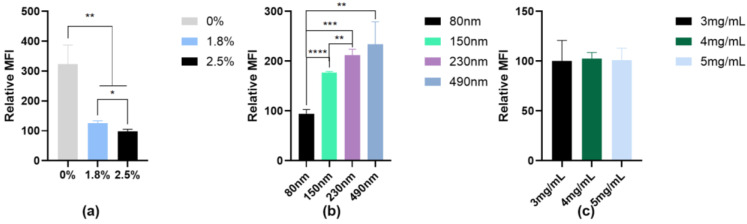
Uptake of IPC-DNVs with different properties by RAW264.7 cells. (**a**) Relative MFI of IPC-DNVs different edge activators amount (0%, 1.8%, 2.5%). (**b**) Relative MFI of IPC-DNVs with different particle sizes (80, 150, 230, 490 nm). (**c**) Relative MFI of IPC-DNVs entrapping increasing concentration of insulin (3, 4, 5 mg/mL). * *p* < 0.05, ** *p* < 0.01, *** *p* < 0.001, **** *p* < 0.0001.

We quantitatively investigated the uptake of IPC-DNVs with different properties by RAW264.7 cells using flow cytometry, and the relative mean fluorescence intensity (MFI) of each group is shown in Figure 8. The uptake results of the different deformability groups showed that the group with 2.5% edge activators (highest deformability) had the lowest relative MFI. Moreover, as the deformability decreased, the relative MFI gradually increased, indicative of increased cell uptake. The relative MFI of the group treated with 0% edge activator was significantly different from those of the other two groups (*p* < 0.01). The uptake results of different particle size groups showed the 80 nm group had a significantly lower MFI than those of the other larger particle size groups. The relative MFI did not exhibit significant differences among the different drug load groups. These trends were consistent with the results obtained using fluorescence microscopy.

### 3.4. Hypoglycemic Effect of IPC-DNVs In Vivo

#### 3.4.1. Impact of Different Properties on the Hypoglycemic Effect of IPC-DNVs In Vivo

**Figure 9:**
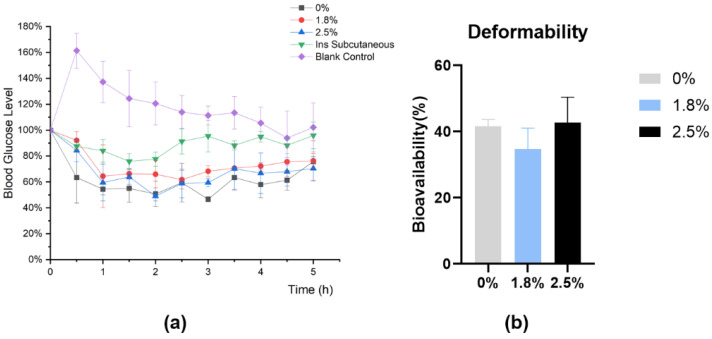
In vivo hypoglycemic effects of IPC-DNVs with edge activators amount (0%, 1.8%, 2.5%). (**a**) Percentage decrease in blood glucose levels. (**b**) Relative bioavailability.

**Figure 10:**
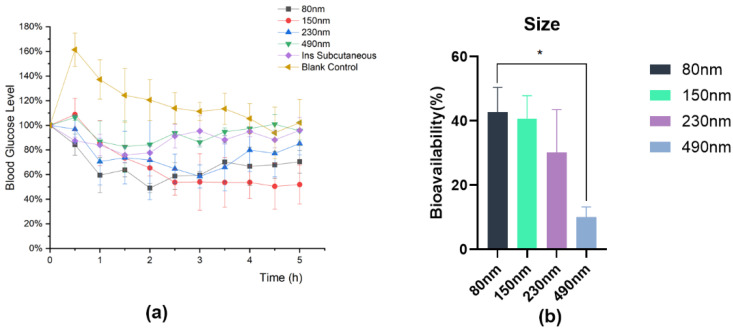
In vivo hypoglycemic effects of IPC-DNVs with different particle sizes (80, 150, 230, 490 nm). (**a**) Percentage decrease in blood glucose levels. (**b**) Relative bioavailability. * *p* < 0.05.

**Figure 11:**
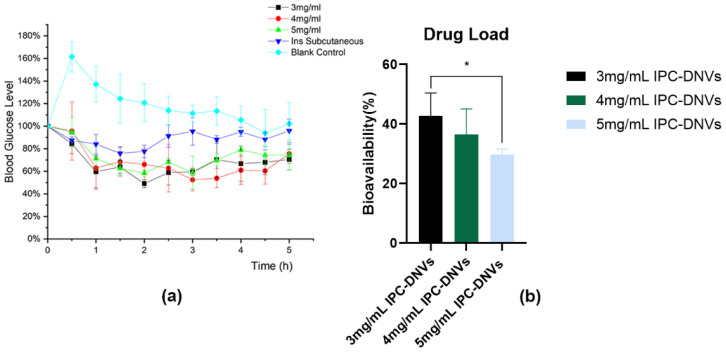
In vivo hypoglycemic effects of IPC-DNVs entrapping increasing concentration of insulin (3, 4, 5 mg/mL). (**a**) Percentage decrease in blood glucose levels. (**b**) Relative bioavailability. * *p* < 0.05.

To ensure accurate dosing and eliminate the impact of IPC-DNV properties on aerosol formation, we used intratracheal instillation for direct administration in the pharmacodynamic experiment. The hypoglycemic effects are shown in Figure 9, Figure 10 and Figure 11. Because of the surgical procedure, there was a spike in blood glucose levels in rats after tracheal exposure, leading to abnormally high initial points in the blood glucose percentage-time curve for the control group. However, in the treatment group, the hypoglycemic effect of the administered drug offset this blood glucose surge.

Different from our expectations, there was no significant difference in the relative bioavailability of IPC-DNVs in different edge activators amount (deformability) groups, and the F values of IPC-DNVs in the 0% (the lowest deformability), 1.8%, and 2.5% groups were 41.65 ± 2.02%, 34.75 ± 6.34%, and 42.72 ± 7.72%, respectively. Additionally, The relative bioavailability of IPC-DNVs in the 80 nm, 150 nm, and 230 nm groups were 42.72 ± 7.72%, 40.76 ± 7.11%, and 30.22 ± 13.30%, respectively, while the relative bioavailability of IPC-DNVs in the 490 nm group was only 10.01 ± 3.22%, showing a significant difference compared with that of the 80 nm group. The relative bioavailability of IPC-DNVs in the 3, 4, and 5 mg/mL groups were 42.72 ± 7.72%, 36.44 ± 8.64%, and 29.74 ± 1.87%, respectively, and there were significant differences between the 3 mg/mL group and the 5 mg/mL group.

#### 3.4.2. Feasibility Verification of the Nebulized Inhalation of IPC-DNVs

**Figure 12:**
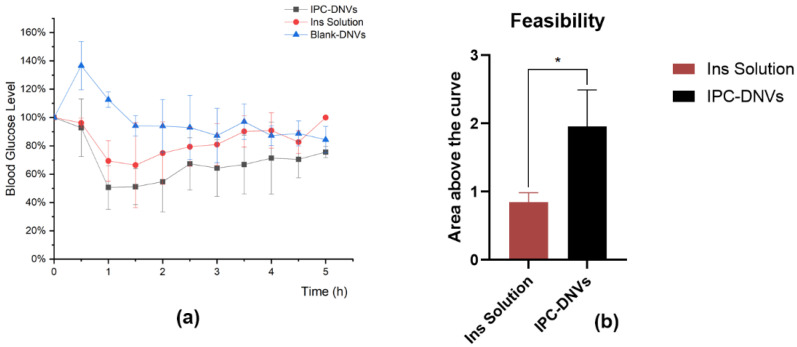
In vivo hypoglycemic effects of IPC-DNVs and insulin solution via nebulized inhalation. (**a**) Percentage decrease in blood glucose levels. (**b**) The area above the blood glucose decline curve with time. * *p* < 0.05.

To better simulate clinical administration, we used nebulized inhalation to compare the hypoglycemic efficacy of IPC-DNVs (edge activators amount, 2.5%; particle size 80 nm; drug load, 3 mg/mL) with insulin solution. The results are shown in Figure 12. Because of the necessity of restraining rats during the administration process, the rats exhibited stress responses and resistance, and there was a spike in blood glucose levels in rats after tracheal exposure, leading to abnormally high initial points in the blood glucose percentage-time curve for the control group. However, in the treatment group, the hypoglycemic effect of the administered drug offset this blood glucose surge. Considering the small tidal volume of rats, a significant portion of the drug aerosol was not inhaled during nebulization treatment, resulting in drug loss Consequently, the dosing amount was uncertain, and F could not be calculated accurately. The results showed that the blood glucose level of the IPC-DNV group was reduced to approximately 50% of the initial level, whereas the blood glucose level in the solution group was reduced to approximately 70% of the initial level. The AAC of the IPC-DNV group was 2.32 times that of the insulin solution group (1.96 ± 0.54 vs. 0.84 ± 0.14).

## 4. Discussion

There were several key considerations in the selection of insulin as a model drug in this study. Insulin, a prototypical biological macromolecule, is an indispensable therapeutic agent for individuals diagnosed with type 1 and severe type 2 diabetes mellitus. However, its high molecular weight, inherent hydrophilicity, and poor stability present serious challenges for devising effective delivery systems, leading to extremely poor patient compliance [37]. To address these issues, there has been significant effort to explore non-injectable insulin delivery methods. The recent successful launch of insulin dry powder formulations demonstrates the viability of transpulmonary biologics delivery [38]. Furthermore, an inherent advantage of using insulin as a model drug is the straightforward assessment of its efficacy, which is directly correlated with changes in blood glucose levels. Thus, we can directly evaluate its therapeutic effect by measuring blood glucose, which is convenient for drug development.

Nebulized inhalation preparations exemplify a combination of pharmaceuticals and medical devices. Drug efficacy is intricately linked to various stages including aerosol production, deposition, clearance, and post-inhalation penetration. Consequently, we identified the pivotal processes governing drug efficacy as evaluation indices. These encompass the aerosolization performance of nebulizer-generated aerosols, in vitro macrophage uptake, and hypoglycemic efficacy after infiltration into the blood. Previous studies have shown that DNVs’ capacity for transmembrane delivery is strongly influenced by both their particle size and deformability [33], and the appropriate particle size and deformability are the basis for crossing the mucosal barrier. The influence of specific quality attributes of inhaled particles, such as size, shape, density, charge, and hydrophilicity, on their interaction with alveolar macrophages, and thus on their fate after inhalation, were also reported [39,40]. Moreover, reducing the volume of nebulized inhalation formulations can enhance patient compliance [41], presenting a challenge for the drug load of the delivery vehicle. Hence, we focused on three key attributes—deformability, particle size, and drug load—to determine vesicular delivery efficacy.

We previously demonstrated the superiority of DNVs for oral mucosal delivery of insulin. The alveolar epithelium, which is thinner than the oral epithelium [42], serves as a crucial barrier for drug delivery from the lungs into systemic circulation. Our results indicated that the introduction of deformability optimized the aerodynamic characteristics of IPC-DNVs. This improvement was likely due to the addition of edge activators, which gradually reduced the surface tension of the IPC-DNVs, facilitating the formation of smaller droplets and more delicate aerosols through the vibrating mesh nebulizers. However, within a certain range (1.8–2.5%), further increase in the amount of edge activator did not significantly enhance the aerodynamic characteristics, suggesting a limit to the influence of surface tension on IPC-DNV atomization. Additionally, introducing deformability reduced the interaction between IPC-DNVs and macrophages. This is consistent with the findings of Sun et al., who showed that deformable liposomes can evade macrophage capture. This resistance to macrophage internalization is likely because deformable nanoparticles remain in contact with the RAW264.7 cell membrane for shorter durations [43]. However, the efficacy of the intratracheal instillation of IPC-DNVs with different deformability varied from our expectations. There was no significant difference in the pharmacodynamic results between the deformable introduction group (2.5% edge activator) and the group without edge activator, despite the former being better suited for membrane crossing. Owing to the elimination of the impact of nebulization performance in intratracheal instillation administration, the main obstacles for the drug to enter the circulation are phagocytosis by alveolar macrophages and the barrier posed by alveolar epithelial cells. Considering the deformability in evading macrophage clearance and the efficacy of tracheal instillation, the advantage of deformable extrusion in transmembrane transport may not be evident during the process of crossing the alveolar epithelium. This indicates that DNVs may predominantly traverse alveolar epithelial cells via a transcellular pathway rather than a paracellular pathway, and may cause partial drug leakage upon entering cells. Overall, introducing deformability optimized the nebulization performance of the vesicles and reduced macrophage phagocytosis, benefiting both localized and systemic drug delivery.

Studies on DNVs for oral mucosal insulin delivery have shown that DNVs within a size range of 80–220 nm achieve better oral absorption [33]. Considering that macrophages are more prone to phagocytosing particles that are larger, we designed target DNVs sizes of 80, 150, 230, and 500 nm for the larger particle size groups. Initially, we attempted to obtain IPC-DNVs of different particle sizes by varying the ultrasonic power. However, the particle size of IPC-DNVs without ultrasonic treatment after hydration was only 200 nm. Thus, IPC-DNVs larger than 200 nm could not be obtained through ultrasonication. Consequently, we employed freeze-drying and reconstitution methods described in our previous study to obtain IPC-DNVs with different particle sizes. The viscosity and surface tension of the atomization solution can affect atomization performance. Excessive viscosity can lead to the blockage of vibrating mesh nebulizers, thereby impairing aerosol generation. Therefore, we screened the effects of differentamount and types of lyophilized protective agents (additives) on the atomization performance of IPC-DNVs. These results indicated that adding more than 8% lyophilized protective agent made it difficult for IPC-DNVs to nebulize. Consequently, we determined that theamount of the lyophilized protective agent should not exceed 8%. There was no apparent difference in atomization performance between the different types of lyophilized protective agents and the control group. Ultimately, we used an 8% trehalose:lactose (4:1) composite protective agent, 5% trehalose:lactose (4:1) composite protective agent, and 8% mannitol to obtain IPC-DNVs with the target particle sizes. The NGI results showed that the particle size did not affect the atomization performance of IPC-DNVs. The uptake of IPC-DNVs of varying particle sizes revealed a notable increase in macrophage uptake with larger particle sizes, whereas IPC-DNVs of approximately 80 nm exhibited minimal engulfment by RAW264.7 cells. This finding was corroborated for IPC-DNVs with different drug load groups (all approximately 80 nm), where significant macrophage uptake was observed when the particle size increased to 150 nm. The uptake results are consistent with the report by Wenhua Wang et al. that particle size is positively correlated with macrophage uptake. This may be because the large and highly active particle surfaces enhance the interaction between the particles and biomolecules on the cell membrane [44]. Additionally, the intratracheal instillation results indicated a gradual decrease in the hypoglycemic efficacy of IPC-DNVs with increasing particle size. This suggests that particle size may influence the penetration of DNVs through the alveolar epithelial cell barrier, with larger particles experiencing greater difficulty crossing the alveolar epithelium. Based on these experimental findings, we propose that for drugs intended for systemic effects via transpulmonary delivery, the particle size of the carrier should be maintained below 150 nm when designing drug delivery systems. However, for topical drugs requiring lung retention, the particle size of the drug delivery systems should be determined based on specific research outcomes considering the combined effects of reduced macrophage phagocytosis and systemic penetration.

The volume of nebulized solution is critical for determining the duration of administration. A prolonged nebulization time can adversely affect patient compliance. Although increasing the drug load can reduce nebulization volume, the capacity of the carrier to encapsulate the drug is limited. High drug load levels may impair carrier stability, leading to drug leakage. In this study, we slightly increased the original drug load and designed three groups of IPC-DNVs at concentrations of 3, 4, and 5 mg/mL. Our findings revealed no significant differences in the nebulization performance or macrophage uptake among DNVs with different drug loadranging from to 3–5 mg/mL. However, the pharmacodynamic results indicated that the DNVs with lower drug load were more effective. This observation suggests that DNVs with lower drug load may play a more important role in entering blood circulation through the alveoli, whereas high drug load DNVs may leak during the transmembrane process, hindering continuous delivery. This conclusion is consistent with our previous findings on oral mucosal delivery [33].

## 5. Conclusions

In this study, we designed a series of IPC-DNVs with varying properties in line with key quality attributes such as deformability, particle size, and drug load. We systematically investigated the impact of these attributes on aerosol atomization performance, macrophage clearance, and bloodstream penetration using both in vitro and in vivo experiments. Our findings indicated that the introduction of deformability enhanced vesicle atomization and decreased macrophage phagocytosis. However, the deformability may not confer distinct advantages to transmembrane penetration of the alveolar epithelium. Although DNV particle size may not directly affect atomization performance, it significantly influenced macrophage uptake and alveolar epithelial barrier penetration. In the range of 80–490 nm, the smaller the particle size of IPC-DNVs, the easier it is to escape phagocytosis by macrophages, and the better the in vivo hypoglycemic efficacy. Notably, variations in the drug load had negligible effects on nebulization performance and macrophage uptake. However, in vivo pharmacodynamic experiments revealed that within the 3–5 mg/mL range, lower drug-loaded DNVs exhibited enhanced drug effects across the epithelium, which may be due to drug leakage during transmembrane processes at higher drug load. Nevertheless, by combining these advantages and administering insulin via nebulized inhalation, the AAC of IPC-DNVs was observed to be 2.32 times higher than that of the insulin solution, demonstrating the feasibility and advantages of DNVs for pulmonary insulin delivery. Our study provides valuable insights and support for the further optimization of DNVs for the pulmonary delivery of biomacromolecule drugs.

## Figures and Tables

**Table 1:** Mean diameter, zeta potential, entrapment efficiency, and deformability index, of IPC-DNVs with edge activators amount (0%, 1.8%, 2.5%). Mean values ± standard deviations are reported.

Amount of Edge Activators	Size (nm)	Zeta Potential (mv)	EE (%)	DI (μg/cm^2^/s)
0%	102.57 ± 0.21	−45.68 ± 0.41	64.04 ± 0.22	6.56 ± 0.66
1.8%	101.57 ± 0.31	−40.18 ± 0.08	79.17 ± 1.71	29.75 ± 0.28
2.5%	83.87 ± 0.71	−35.85 ± 0.56	83.97 ± 0.54	39.30 ± 0.99

**Table 2:** Mean diameter, zeta potential, and entrapment efficiency of IPC-DNVs with different particle sizes (80, 150, 230, 490 nm). Mean values ± standard deviations are reported.

Target Size	Size (nm)	Zeta Potential (mv)	EE (%)
80 nm	83.87 ± 0.71	−35.85 ± 0.56	83.97 ± 0.54
150 nm	150.60 ± 1.55	−47.33 ± 0.32	86.926 ± 0.18
230 nm	238.70 ± 1.80	−46.13 ± 0.08	88.06 ± 2.64
500 nm	477.83 ± 11.21	−49.09 ± 0.31	88.31 ± 4.54

**Table 3:** Mean diameter, zeta potential, and entrapment efficiency of IPC-DNVs entrapping increasing concentration of insulin (3, 4, 5 mg/mL). Mean values ± standard deviations are reported.

Drug Load	Size (nm)	Zeta Potential (mv)	EE (%)
3 mg/mL	83.87 ± 0.71	−35.85 ± 0.56	83.97 ± 0.54
4 mg/mL	89.13 ± 0.15	−36.04 ± 0.43	84.47 ± 0.43
5 mg/mL	85.00 ± 0.40	−35.00 ± 0.38	82.85 ± 5.59

## Data Availability

The datasets used and/or analyzed during the current study are available from the corresponding author upon reasonable request.

## Supplementary Materials

The following supporting information can be downloaded at: https://www.mdpi.com/article/10.3390/pharmaceutics16070879/s1, Figure S1: The actual deposition percentages of insulin at each next generation impactor stage of IPC-DNVs with edge activators amount (0%, 1.8%, 2.5%); Figure S2: The actual deposition percentages of insulin at each next generation impactor stage of IPC-DNVs with different particle sizes (80, 150, 230, 490 nm); Figure S3: The actual deposition percentages of insulin at each next generation impactor stage of IPC-DNVs entrapping increasing concentration of insulin (3, 4, 5 mg/mL).

## Abbreviations

DNVs: deformable nanovesicle; IPC-DNVs, deformable nanovesicles loaded with insulin-phospholipid complex; AAC, the area above the blood glucose decline curve with time; MMAD, mass median aerodynamic diameter; PBS, phosphate-buffered saline; PDI, polydispersity index; HPLC, high performance liquid chromatography; FPF, fine particle fraction; GSD, geometric standard deviation; NGI, next-generation impactor; MFI, mean fluorescence intensity; EE, entrapment efficiency; DI, deformability index.

## References

[1] Peng S., Wang W., Zhang R., Wu C., Pan X., Huang Z. (2024). Nano-Formulations for Pulmonary Delivery: Past, Present, and Future Perspectives. Pharmaceutics.

[2] Feng X., Shi Y., Zhang Y., Lei F., Ren R., Tang X. (2024). Opportunities and Challenges for Inhalable Nanomedicine Formulations in Respiratory Diseases: A Review. Int. J. Nanomed..

[3] Viegas C., Patrício A.B., Prata J.M., Nadhman A., Chintamaneni P.K., Fonte P. (2023). Solid Lipid Nanoparticles vs. Nanostructured Lipid Carriers: A Comparative Review. Pharmaceutics.

[4] Li D., Zhao A., Zhu J., Wang C., Shen J., Zheng Z., Pan F., Liu Z., Chen Q., Yang Y. (2023). Inhaled Lipid Nanoparticles Alleviate Established Pulmonary Fibrosis. Small.

[5] Shirley M. (2019). Amikacin Liposome Inhalation Suspension: A Review in Mycobacterium avium Complex Lung Disease. Drugs.

[6] Abdel-Hafez S.M., Hathout R.M., Sammour O.A. (2018). Curcumin-loaded ultradeformable nanovesicles as a potential delivery system for breast cancer therapy. Colloids Surf. B Biointerfaces.

[7] Guo Y., Yang Y., Xu Y., Meng Y., Ye J., Xia X., Liu Y. (2022). Deformable Nanovesicle-Loaded Gel for Buccal Insulin Delivery. Pharmaceutics.

[8] Aundhia C., Shah N., Talele C., Zanwar A., Kumari M., Patil S. (2024). Enhancing Gene Therapy through Ultradeformable Vesicles for Efficient siRNA Delivery. Pharm. Nanotechnol..

[9] Khan I., Needham R., Yousaf S., Houacine C., Islam Y., Bnyan R., Sadozai S.K., Elrayess M.A., Elhissi A. (2021). Impact of phospholipids, surfactants and cholesterol selection on the performance of transfersomes vesicles using medical nebulizers for pulmonary drug delivery. J. Drug Deliv. Sci. Technol..

[10] Qin L., Cui Z., Wu Y., Wang H., Zhang X., Guan J., Mao S. (2022). Challenges and Strategies to Enhance the Systemic Absorption of Inhaled Peptides and Proteins. Pharm. Res..

[11] Yong J., Shu H., Zhang X., Yang K., Luo G., Yu L., Li J., Huang H. (2024). Natural Products-Based Inhaled Formulations for Treating Pulmonary Diseases. Int. J. Nanomed..

[12] Mei X., Li J., Wang Z., Zhu D., Huang K., Hu S., Popowski K.D., Cheng K. (2023). An inhaled bioadhesive hydrogel to shield non-human primates from SARS-CoV-2 infection. Nat. Mater..

[13] Han M.-M., Tang L., Huang B., Li X.-N., Fang Y., Qi L., Duan B., Yao Y., He Y., Xing L. (2024). Inhaled nanoparticles for treating idiopathic pulmonary fibrosis by inhibiting honeycomb cyst and alveoli interstitium remodeling. J. Control. Release.

[14] Zhang T., Chen Y., Ge Y., Hu Y., Li M., Jin Y. (2018). Inhalation treatment of primary lung cancer using liposomal curcumin dry powder inhalers. Acta Pharm. Sin. B.

[15] Lin X., Kankala R.K., Tang N., Xu P., Hao L., Yang D., Wang S., Zhang Y.S., Chen A. (2018). Supercritical Fluid-Assisted Porous Microspheres for Efficient Delivery of Insulin and Inhalation Therapy of Diabetes. Adv. Heal. Mater..

[16] Fröhlich E., Salar-Behzadi S. (2021). Oral inhalation for delivery of proteins and peptides to the lungs. Eur. J. Pharm. Biopharm..

[17] Tsao C., Yuan Z., Zhang P., Liu E., McMullen P., Wu K., Hung H.-C., Jiang S. (2020). Enhanced pulmonary systemic delivery of protein drugs via zwitterionic polymer conjugation. J. Control. Release.

[18] Ceschan N.E., Scioli-Montoto S., Sbaraglini M.L., Ruiz M.E., Smyth H.D., Bucalá V., Ramírez-Rigo M.V. (2021). Nebulization of a polyelectrolyte-drug system for systemic hypertension treatment. Eur. J. Pharm. Sci..

[19] Ponkshe P., Wang Y., Tan C. (2023). Systemic Protein Delivery via Inhalable Liposomes: Formulation and Pharmacokinetics. Pharmaceutics.

[20] Adorni G., Seifert G., Buttini F., Colombo G., Stecanella L.A., Krämer I., Rossi A. (2019). Aerosolization Performance of Jet Nebulizers and Biopharmaceutical Aspects. Pharmaceutics.

[21] Troy M., Van Vleet J., Tashkin D., Barjaktarevic I. (2023). Recent advances predict a bright future for nebulizers. Curr. Opin. Pulm. Med..

[22] Pritchard J.N., Hatley R.H., Denyer J., von Hollen D. (2018). Mesh nebulizers have become the first choice for new nebulized pharmaceutical drug developments. Ther. Deliv..

[23] Falahati M., Hasan A., Zeinabad H.A., Serpooshan V., von der Thüsen J., Hagen T.L.T. (2023). Engineering of pulmonary surfactant corona on inhaled nanoparticles to operate in the lung system. Nano Today.

[24] Ho D.-K., Nichols B.L., Edgar K.J., Murgia X., Loretz B., Lehr C.-M. (2019). Challenges and strategies in drug delivery systems for treatment of pulmonary infections. Eur. J. Pharm. Biopharm..

[25] Lee W.T., Lee H., Kim J., Jung Y., Choi E., Jeong J.H., Jeong J.-H., Lee J.H., Youn Y.S. (2024). Alveolar macrophage phagocytosis-evading inhaled microgels incorporating nintedanib-PLGA nanoparticles and pirfenidone-liposomes for improved treatment of pulmonary fibrosis. Bioact. Mater..

[26] Subramanian S., Khan I., Korale O., Alhnan M.A., Ahmed W., Najlah M., Taylor K.M., Elhissi A. (2016). A simple approach to predict the stability of phospholipid vesicles to nebulization without performing aerosolization studies. Int. J. Pharm..

[27] Khan I., Apostolou M., Bnyan R., Houacine C., Elhissi A., Yousaf S.S. (2020). Paclitaxel-loaded micro or nano transfersome formulation into novel tablets for pulmonary drug delivery *via* nebulization. Int. J. Pharm..

[28] Liu Q., Zhang X., Xue J., Chai J., Qin L., Guan J., Zhang X., Mao S. (2022). Exploring the intrinsic micro-/nanoparticle size on their in vivo fate after lung delivery. J. Control. Release.

[29] Zhao J., Qin L., Song R., Su J., Yuan Y., Zhang X., Mao S. (2022). Elucidating inhaled liposome surface charge on its interaction with biological barriers in the lung. Eur. J. Pharm. Biopharm..

[30] Zhao J., Su J., Qin L., Zhang X., Mao S. (2020). Exploring the influence of inhaled liposome membrane fluidity on its interaction with pulmonary physiological barriers. Biomater. Sci..

[31] Liu Q., Xue J., Zhang X., Chai J., Qin L., Guan J., Zhang X., Mao S. (2022). The influence of a biomimetic pulmonary surfactant modification on the in vivo fate of nanoparticles in the lung. Acta Biomater..

[32] Xu Y., Zhang X., Zhang Y., Ye J., Wang H.-L., Xia X., Liu Y. (2018). Mechanisms of deformable nanovesicles based on insulin-phospholipid complex for enhancing buccal delivery of insulin. Int. J. Nanomed..

[33] Yang Y., Guo Y., Xu Y., Meng Y., Zhang X., Xia X., Liu Y. (2020). Factors affecting the buccal delivery of deformable nanovesicles based on insulin–phospholipid complex: An *in vivo* investigation. Drug Deliv..

[34] Liu J., Gong T., Fu H., Wang C., Wang X., Chen Q., Zhang Q., He Q., Zhang Z. (2008). Solid lipid nanoparticles for pulmonary delivery of insulin. Int. J. Pharm..

[35] Hu J., Zhang R., Beng H., Deng L., Ke Q., Tan W. (2019). Effects of flow pattern, device and formulation on particle size distribution of nebulized aerosol. Int. J. Pharm..

[36] Lombry C., Edwards D.A., Préat V., Vanbever R. (2004). Alveolar macrophages are a primary barrier to pulmonary absorption of macromolecules. Am. J. Physiol. Cell. Mol. Physiol..

[37] Wang M., Wang C., Ren S., Pan J., Wang Y., Shen Y., Zeng Z., Cui H., Zhao X. (2022). Versatile Oral Insulin Delivery Nanosystems: From Materials to Nanostructures. Int. J. Mol. Sci..

[38] Dailey G., Ahmad A., Polsky S., Shah V. (2016). A novel option for prandial insulin therapy: Inhaled insulin. Postgrad. Med..

[39] Patel B., Gupta N., Ahsan F. (2015). Particle engineering to enhance or lessen particle uptake by alveolar macrophages and to in-fluence the therapeutic outcome. Eur. J. Pharm. Biopharm..

[40] Soni S.S., Kim K.M., Sarkar B., Rodell C.B. (2024). Uptake of Cyclodextrin Nanoparticles by Macrophages is Dependent on Particle Size and Receptor-Mediated Interactions. ACS Appl. Bio Mater..

[41] Flament M., Leterme P., Burnouf T., Gayot A. (1997). Jet nebulisation: Influence of dynamic conditions and nebuliser on nebulisation quality. Application to the α 1 protease inhibitor. Int. J. Pharm..

[42] Ngan C.L., Asmawi A.A. (2018). Lipid-based pulmonary delivery system: A review and future considerations of formulation strategies and limitations. Drug Deliv. Transl. Res..

[43] Sun D., Zhang G., Xie M., Wang Y., Liang X., Tu M., Su Z., Zeng R. (2023). Softness enhanced macrophage-mediated therapy of inhaled apoptotic-cell-inspired nanosystems for acute lung injury. J. Nanobiotechnol..

[44] Wang W., Yang B., Huang Z., Huang Y., Hu P., Pan X., Wu C. (2021). Investigating the Effect of Particle Size on Cellular Uptake by Aggregation-Caused Quenching Probe–Encapsulating Solid Lipid Nanoparticles, Inhaled. J. Pharm. Innov..

