# Supplementary material for: Investigation of Factors Influencing the Effectiveness of Deformable Nanovesicles for Insulin Nebulization Inhalation
Source: Pharmaceutics. 2024 Jun 29;16(7):879. doi: 10.3390/pharmaceutics16070879 (PMC11280345; doi:10.3390/pharmaceutics16070879)
Supplement: Supplementary file 1 [file pharmaceutics-16-00879-s001.zip › pharmaceutics-3048843-supplementary.pdf]

Supplementary Material

Investigation of Factors Influencing the Effectiveness of De-formable Nanovesicles for Insulin Nebulization Inhalation

Jinghan Yu [1], Yingying Meng [1], Zhiyang Wen [1], Yu Jiang [1], Yiyue Guo [2], Simeng Du [1], Yuling Liu [1,*] and Xuejun Xia [1,*]

[1] Beijing Key Laboratory of Drug Delivery Technology and Novel Formulations, State Key Laboratory of Bioactive Substance and Function of Natural Medicines, Department of Pharmaceutics, Institute of Materia Medica, Chinese Academy of Medical Sciences & Peking Union Medical College, Beijing 100050, China; yujinghan@imm.ac.cn (J.Y.); mengyingying@imm.ac.cn (Y.M.); wenzhiyang@imm.ac.cn (Z.W.); yu.jiang@wehandbio.com (Y.J.); dusimeng@imm.ac.cn (S.D.)

[2] Beijing Wehand-Bio Pharmaceutical Co., Ltd., Beijing 102600, China; yiyue.guo@wehandbio.com

* Correspondence: ylliu@imm.ac.cn (Y.L.); xjxia@imm.ac.cn (X.X.)

1. Figures

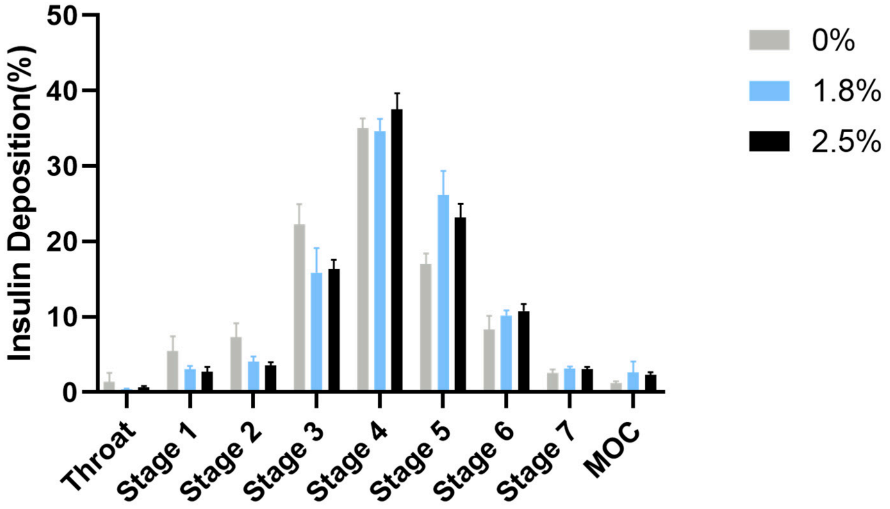

Figure S1. The actual deposition percentages of insulin at each next generation impactor stage of IPC-DNVs with edge activators amount (0%, 1.8%, 2.5%).

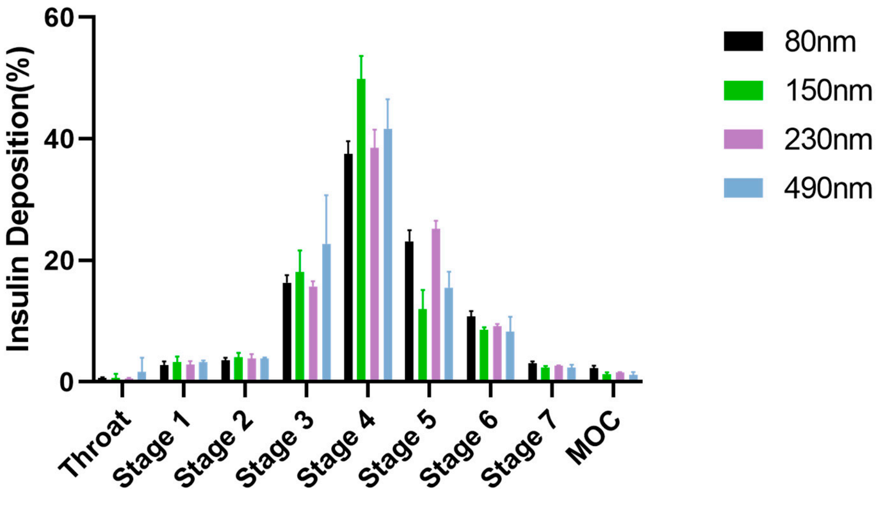

Figure S2. The actual deposition percentages of insulin at each next generation impactor stage of IPC-DNVs with different particle sizes (80, 150, 230, 490 nm).

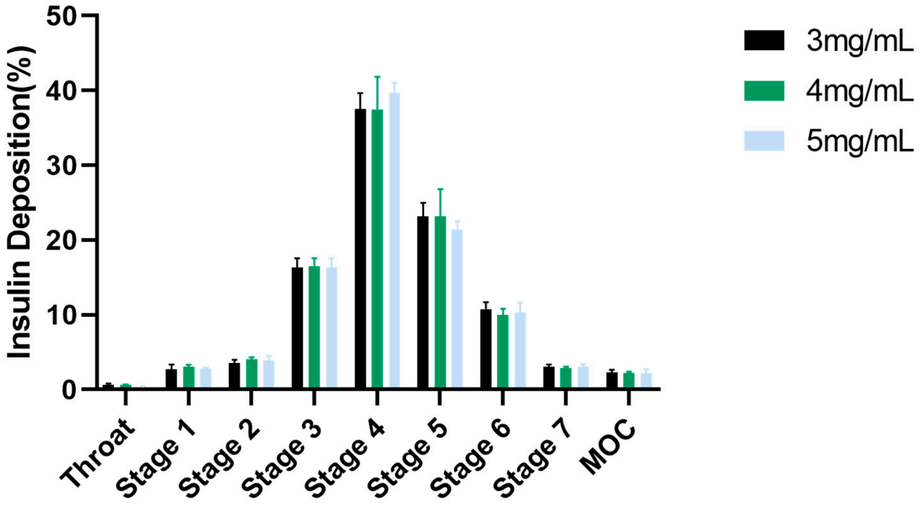

Figure S3. The actual deposition percentages of insulin at each next generation impactor stage of IPC-DNVs entrapping increasing concentration of insulin (3, 4, 5 mg/mL).
